# On the unusual amber coloration of nanoporous sol-gel processed Al-doped silica glass: An experimental study

**DOI:** 10.1038/s41598-019-48917-4

**Published:** 2019-08-28

**Authors:** Alvin Chang, Yujuan He, Maria A. Torres Arango, Maoyu Wang, Yang Ren, Zhenxing Feng, Chih-Hung Chang, Konstantinos A. Sierros

**Affiliations:** 1 School of Chemical, Biological and Environmental Engineering, Oregon State University, Corvallis, OR 97331 USA; 2 Mechanical & Aerospace Engineering, West Virginia University, Morgantown, WV 26506 – 6106 USA; 3 Advanced Photon Source, Argonne National Laboratory, 9700S Cass Avenue Argonne, Chicago, IL 60439 USA

**Keywords:** Materials science, Materials for optics

## Abstract

Silica is the most abundant component on the earth’s surface. It plays an important role in many natural processes. Silica is also a critical material for a wide range of technical applications such as in optics and electronics. In this work, we discuss our recent experimental observation of the unusual amber coloration of aluminum doped sol-gel glass that has not been reported in the past. We characterized Al-doped sol-gel glasses, prepared at different sintering temperature, using a plethora of techniques to investigate the origin of this unusual coloration and to understand their structural and chemical properties. We used these experimental results to test a number of possible coloring mechanisms. The results suggested this coloring is likely caused by temperature-dependent aluminum-associated defect centers associated with different amorphous-to-crystalline ratios of the annealed sol-gel silica glass structures.

## Introduction

Silica occurs in nature in many forms and is a key component of the earth’s crust and mantle. It plays an important role in many geological^1^ and biological processes^2^. In industry, silica is an important material for a wide range of applications^3^ including optics, electronics, catalysts, sorbents, and as fillers for many products such as paints, rubbers, and roadways. Thus, the properties of silica are of fundamental interest in many areas. Silica can be synthesized via different approaches to produce products in various forms including fused silica^4^, fumed silica^1,5^, silica gel^6^, and aerogels^7,8^. As demonstrated by several groups, the sol-gel method is a unique technique to produce silica that is difficult to achieve by other processes such as melting^9–11^.

In this study, we report on the discovery of an unusual amber coloration of nanoporous sol-gel based silica glass when doped with Aluminum (Al). In particular, Al-doped silica glass was prepared via a sol-gel method. Surprisingly, the obtained glass exhibited colors ranging from clear, light amber, dark brown, and back to clear again at different thermal annealing temperatures. Aluminum is a known dopant that alters silica properties such as melt rheology^12^, corrosion resistance^13^, and molecular diffusivity of oxygen^14^. The effects of aluminum dopant on density, refractive index, and ultrasonic transmission of silica glasses have been reported in the literature^15^. However, we could not find reports of this unusual sintering-temperature dependent amber coloration of sol-gel glass in the literature.

Colored glasses find several important applications in art and technology. Early glass coloring techniques trace their origin back to ancient Egypt and Rome^16^. Nowadays, a variety of techniques with different color producing mechanisms are available to render colors in glass^17^. For example, the addition of iron oxides or iron polysulfides can produce bluish-green and amber colored glass, respectively^18^. Green and amber colored glasses are key materials to the manufacturing of food and beverage bottles for maintaining freshness and long-lasting taste^19^.

Another technique to produce colored glass employs the addition of nanoparticles. Adding small amounts of gold can produce ruby-gold glass, which is arguably the most beautiful and celebrated colored glass. In this case, the intense red color originates from the dispersed plasmonic-resonant gold nanoparticles^20^. As such, the use of light scattering is an alternative approach to yield color in the glass. Phase separated glasses exhibit an opaque color due to diffuse light scattering caused by the difference in the refractive index of each phase. Tomioka *et al*.^21^ investigated the phase separation behavior of multicomponent oxide glasses and observed that the different microstructures of phase separation resulted in a whitish to a bluish color. Another approach is to create three-dimensional photonic structures to render noniridescent structural colors. Schroden *et al*.^22^ prepared inverse opal photonic crystals of silica using ordered arrays of uniformly sized polymer spheres infiltrated with silica fluid precursors. The color characteristics, physical and chemical properties, and cost of manufacturing vary significantly between these different approaches. However, many coloring mechanisms are still not well understood. To effectively use the suitable approaches for various applications, it is important to understand their structure and process relationships. To elucidate the origin of this unusual coloration and to understand their structural and chemical properties, we characterized Al-doped sol-gel glasses prepared at different sintering temperature using various techniques. We used these experimental results to test a number of possible coloring mechanisms.

## Results and Discussion

**Figure 1:**
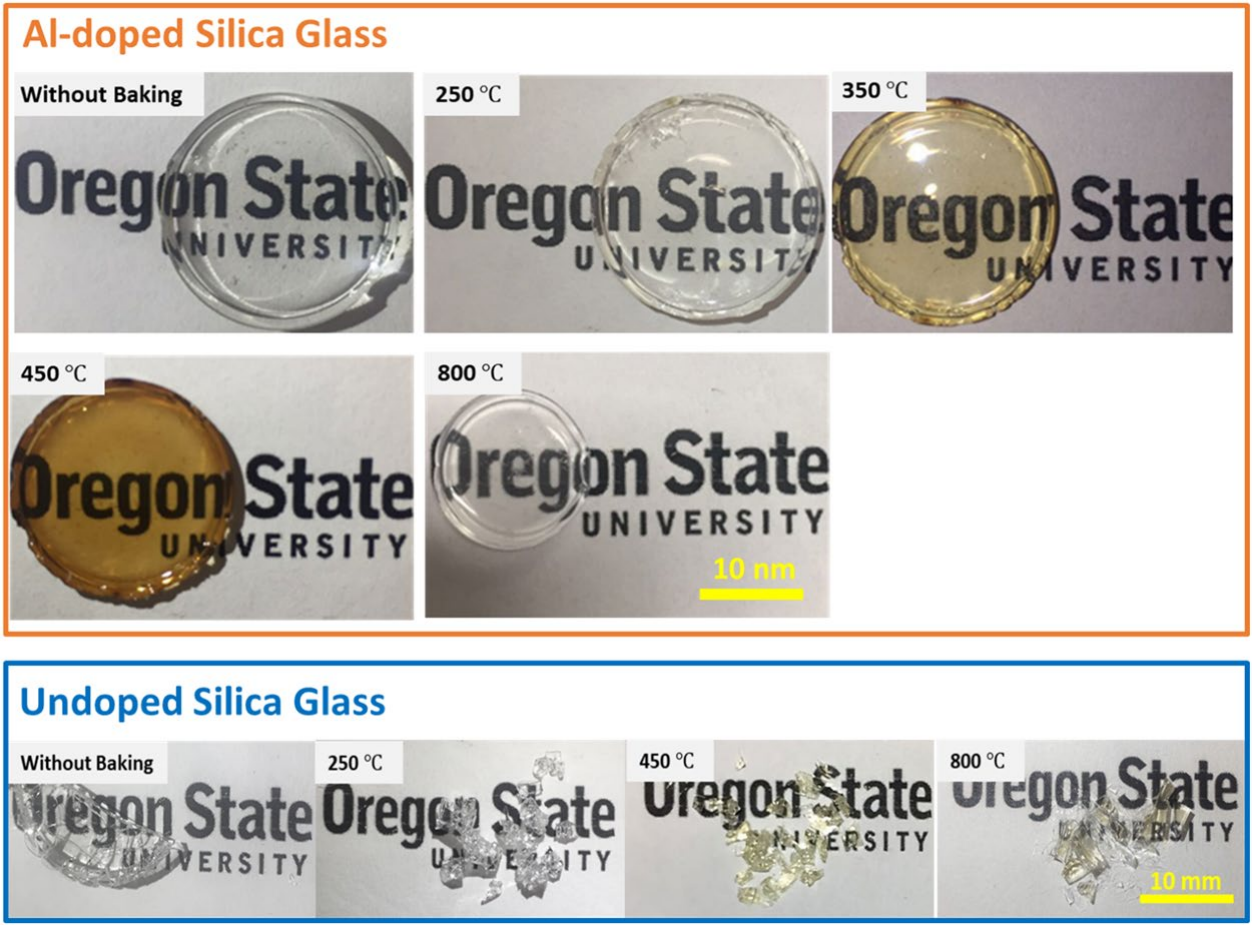
(Top) Al-doped silica sol-gel glass samples prepared with and without annealing exhibiting temperature-dependent clear and amber coloring. (Bottom) Undoped silica glass is depicted for comparison purposes.

**Figure 2:**
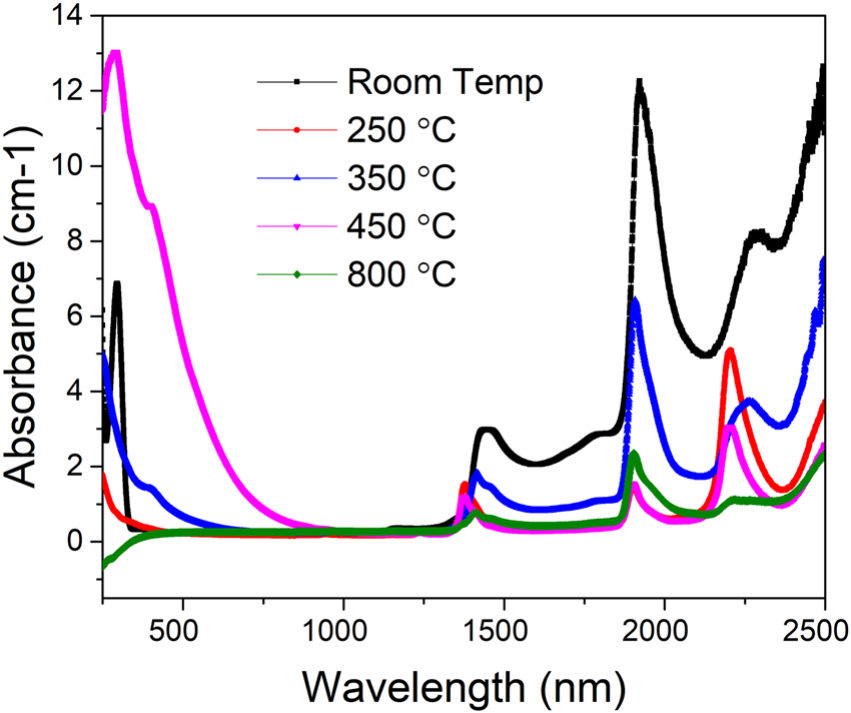
Absorption spectra of the samples annealed at different temperatures.

Figure 1 depicts the coloration stages of Al-doped and undoped Silica glass along with optical transmittance data ranging from UV to Visible to IR light for various annealing temperatures. For the Al-doped silica materials, the color ranges from clear when there is no annealing and when annealed to 250 °C, to light amber at 350 °C, and to dark amber at 450 °C as shown in Fig. 1. At 800 °C the glass coloration returns to a clear and optically transparent color. There is no such coloration range, nor are there even any color changes observed for the undoped silica glass annealed at the same temperature range. The sample annealed at 800 °C exhibits a negative absorbance in the UV region (Fig. 2) which is due to its photoluminescent emission (S5 & 7, Support Material) and may be attributed to the Al dopant^19^. This is not observed for the other samples annealed at different temperatures and highlights the potential role that the dopant plays in the coloration process. We cannot obtain monolithic glass from the undoped silica gel as the aluminum is needed to strengthen the glass.

**Figure 3:**
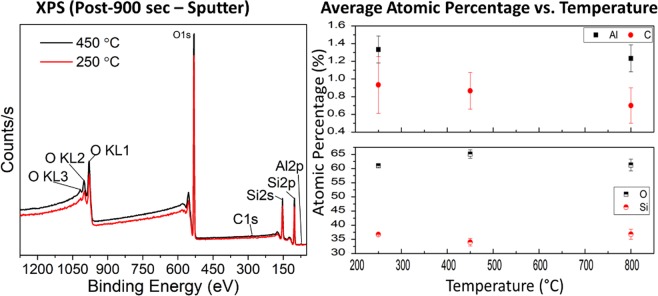
(Left) X-ray photoelectron spectroscopy spectra for 250 °C and 450 °C annealed glass. (Right) Average atomic percentage vs. temperature for Al, C, O, and Si elements.

In order to understand the uncommon amber coloration, it is important to investigate chemical and structural properties of the prepared sol-gel glass system as plausible causes. An initial approach is to hypothesize that the potential formation of carbon (C), elemental composition differences formed by annealing, and/or contamination of the sol-gel glass with other elements such as Fe and S can render an amber color with temperature dependence^23^. For this study, we employed X-ray photoelectron spectroscopy (XPS) to test these hypotheses. Figure 3 (left) shows the XPS survey spectra for 250 °C and 450 °C annealed glass, respectively. Presence of Si 2p and O 1s peaks are observed. However, there is a negligible amount of adventitious C detected. Usually, adventitious C contamination is expected to include C-C, C-O-C, and O-C=O components at around 284.8 eV with some C layer formation appearing even at 286 eV for the native oxide of Al^24^. Figure 3 (right) depicts the atomic percentage change of Al, C, O and Si with annealing temperature. There is very little elemental composition difference found for all elements as annealing temperature increases. In particular, the average atomic percentage of C is found to be below 1% in all cases, as shown by Fig. 3 (right). Therefore, the formation of C or any significant composition differences and contamination by other elements can be excluded as a possible reason for the amber coloration.

**Figure 4:**
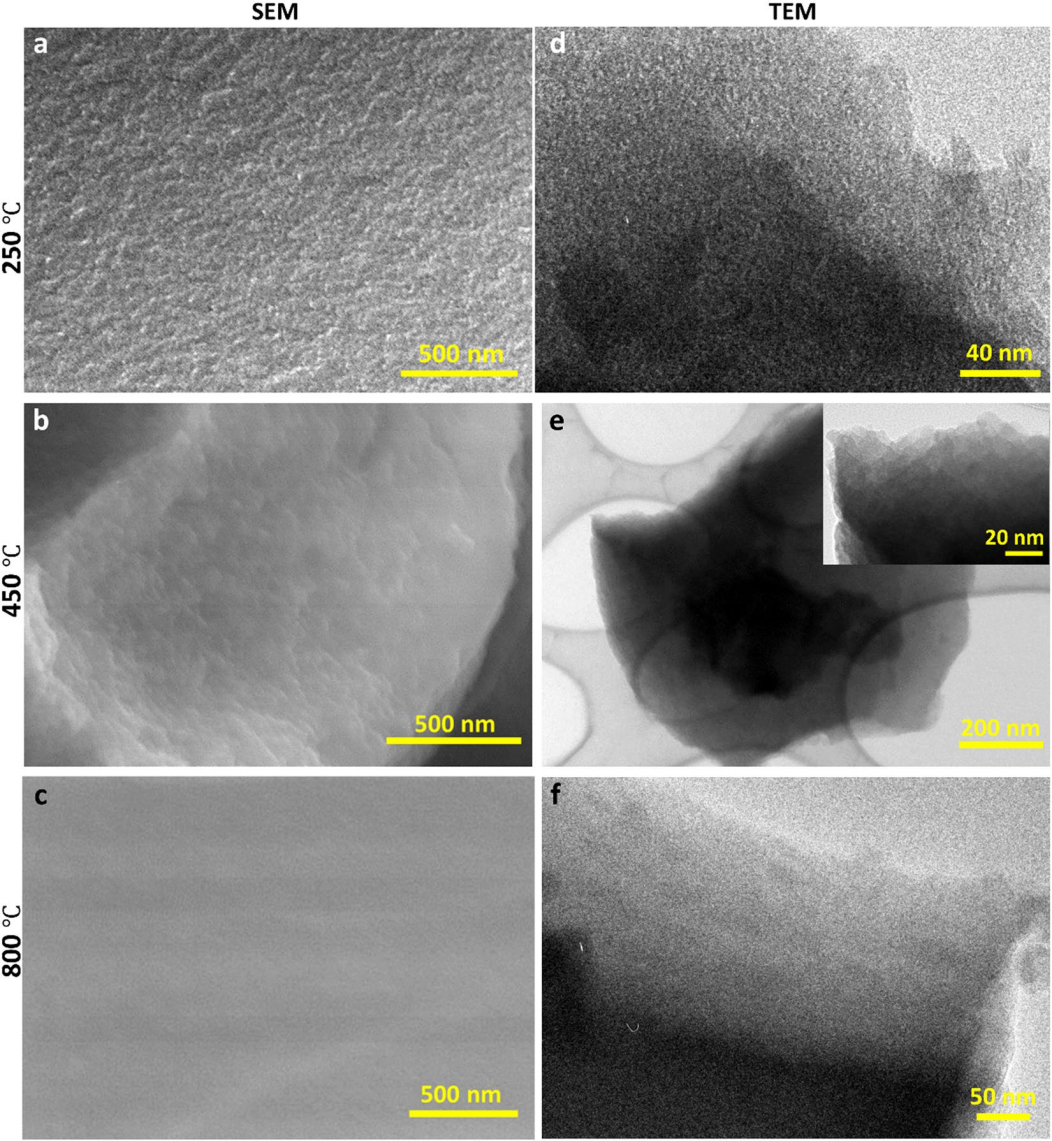
The SEM (**a**–**c**) and TEM (**d**–**f**) images of the glass samples heated to 250 °C, 450 °C, and 800 °C, respectively.

**Figure 5:**
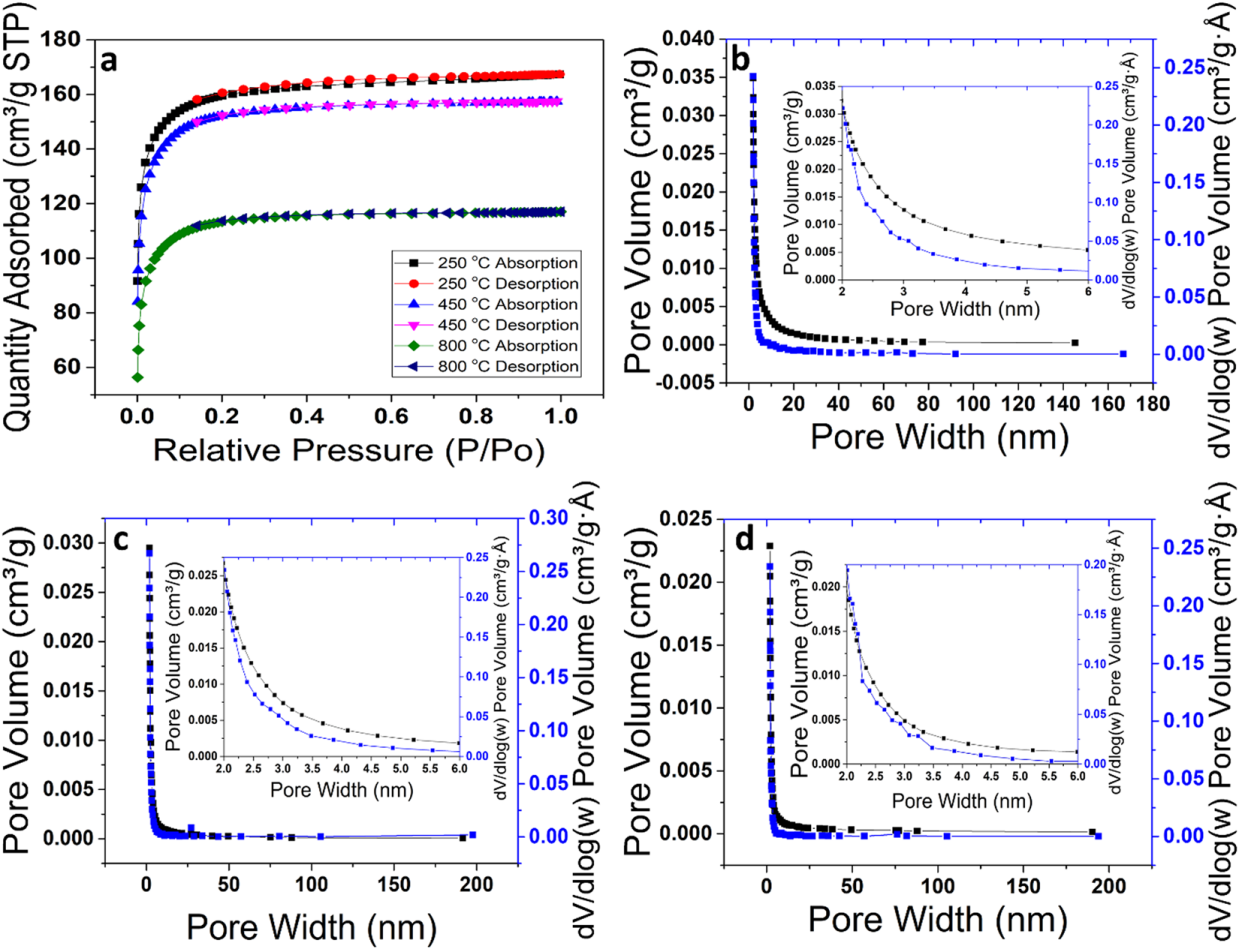
**a**) Nitrogen adsorption-desorption isotherm of the glass samples heated to the different temperatures. The Barrett- Joyner-Halenda (BJH) pore size distribution plot of the glass samples heated to (**b**) 250 °C, (**c**) 450 °C, and (**d**) 800 °C.

Another plausible cause for the unusual coloration may be the interaction of porous glass microstructures with incident light. This phenomenon of tunable structural coloring can be observed in many biological systems such as in butterfly wings^25^ and plants^26^. In this case; we hypothesize that an amorphous array of air pores with short-range order forming in the structure leads to the unusual amber color. In order to test this hypothesis, we conducted porosimetry, scanning electron microscopy (SEM), and transmission electron microscopy (TEM) experiments, as shown in Fig. 4. Barrett-Joyner-Halenda (BJH)^27^ N adsorption measurements (Fig. 5) suggest that formed pores lie in the micro-pore range (i.e., between 0 and 2 nm). Furthermore, the pore size decreased with increased annealing temperature. SEM and TEM measurements further validate the porosimetry data. Such size range clearly does not produce structural color in the visible range^28^.

**Figure 6:**
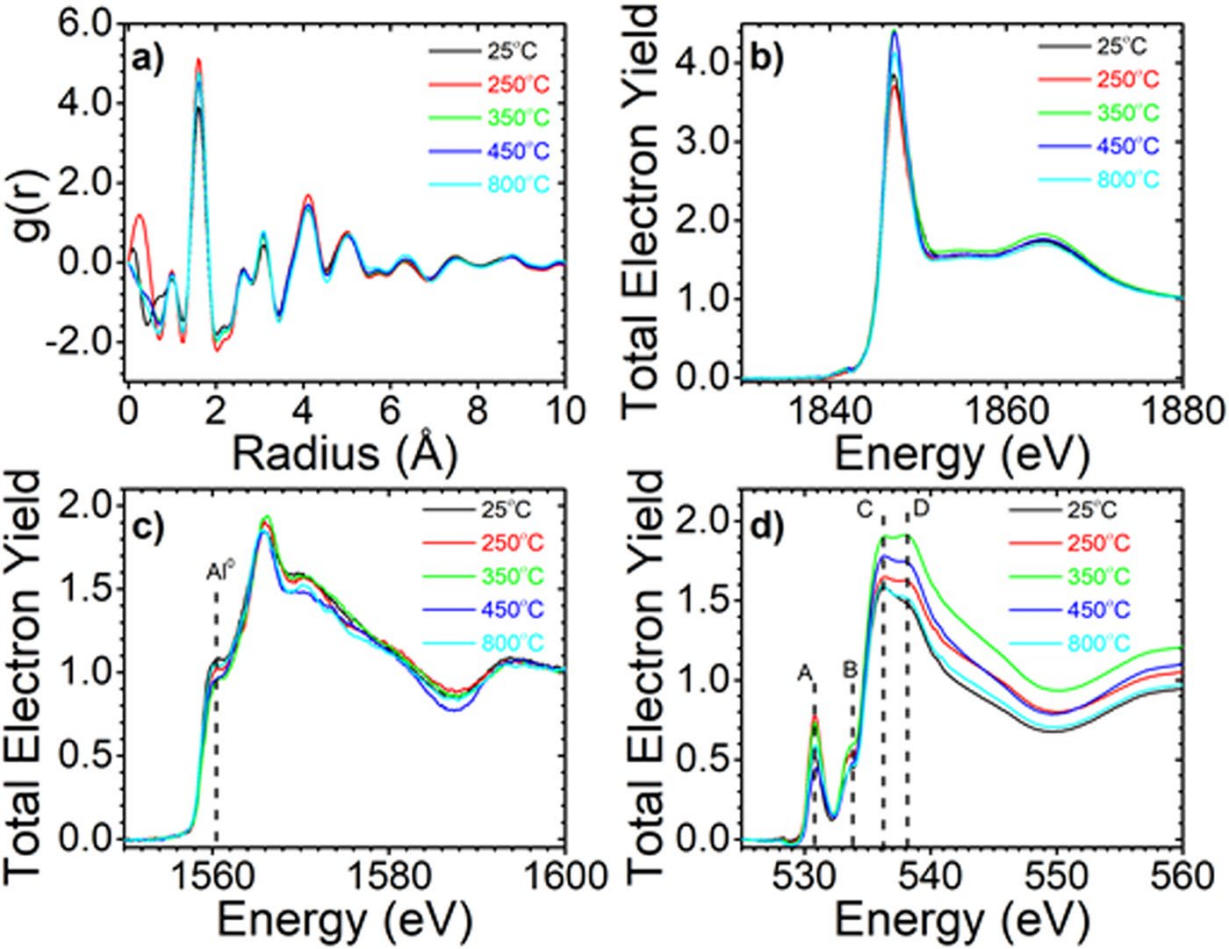
(**a**) Pair distribution function (PDF) data and X-ray absorption spectroscopy data at the (**b**) Si K-edge (**c**) Al K-edge, and (**d**) O K-edge for Al-doped Si glass at various annealing temperatures.

To explore the possible structural changes that may be associated with the amber glass coloration, Pair Distribution Function - PDF (Fig. 6a) was conducted on these Al-doped glasses annealed at various temperatures. As shown in Fig. 6a, the PDF spectra shows similar patterns for an amorphous Si glass, suggesting that ensemble-averaged local structures are not responsible for the coloration in amber glasses. However, some differences were found for element-specific XAS spectra. Figure 6b is the Si K-edge XAS around 1848 eV^29^. It shows that all Al-doped Si glasses are in the same amorphous phase as SiO_2_, which eliminates the phase transition influence^30^. In contrast, Al and O XAS spectra in Fig. 6c,d, respectively, exhibit temperature-dependent changes. It is noted that Al doped in amber glasses are mostly in the metallic state as featured by a peak at 1560 eV^29,31^, and that the metallic Al peak intensity decreases as temperature increases. Interestingly, the Al^0^ peak (represented by the dashed line in Fig. 6c) intensities of amber glass annealed at 350 °C and 450 °C are the lowest, indicating the existence of aluminum oxide (Al_2_O_3_) that may introduce defects in the SiO_2_ unit cell.

Those defects are also suggested in O K-edge XAS (Fig. 6d). Peak B (Fig. 6d) around 534 eV is assigned to the unoccupied O states of SiO_x_^32^, which show the similar temperature-dependent trend as that of the metallic Al^0^ peak in Fig. 6c: a higher peak B (more unoccupied O states) when temperature raises up to 350 °C, and a much lower peak B (less unoccupied O states) when the temperature increases to 800 °C. In addition, peaks C and D (Fig. 6d) represent the existence of single crystalline and amorphous SiO_x_^33^. It also shows the same variation trend as that of unoccupied O states and the metallic Al° peak. Therefore, annealing temperature affects the amount of Al atoms doped into the SiO_2_ unit cell, which results in different oxygen defects and different degrees of amorphous/crystalline ratio due to the various valence state of Al and Si. A considerable amount of studies on point defects in crystalline silica (i.e., quartz) have been conducted due to the important role of quartz in nature and its many technical applications^34–48^. These point defects are either structural related or impurity related defects, including the [AlO_4_]°, and [AlO_4_/H^+^] centers, oxygen, and oxygen vacancy-related defect centers^35,36^. The aluminum-associated hole center, [AlO_4_]°_,_ is believed to cause the “smoky” coloration of quartz crystals. Griffiths *et al*. reported the paramagnetic centers with Al hyperfine structure in irradiated and natural “smoky” α-quartz first^37^. Since then many research groups have investigated the aluminum-associated hole centers in “smoky” quartz crystals^38–48^. Subjecting synthetic α-quartz to high energy electrons radiation, Koumvakalis introduced an optical absorption in the visible range and attributed it to the aluminum-associated hole center via simultaneous optical absorption and ESR measurements^45^. Our data suggest that the unusual amber coloration from the aluminum-doped sol-gel silica glass could be associated with the aluminum-associated hole centers.

**Figure 7:**
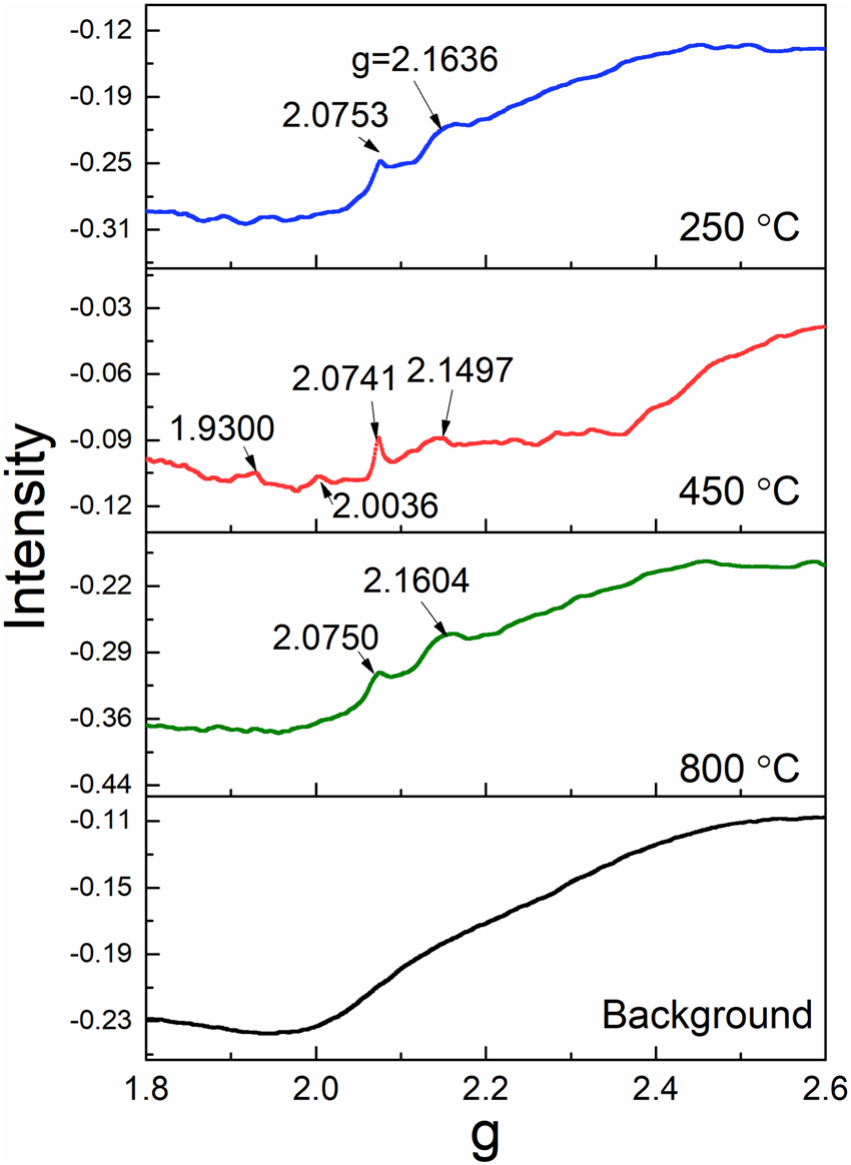
EPR data of the samples annealed at different temperatures.

EPR experiments were performed on glass annealed to 250 °C, 450 °C, and 800 °C, and the results are presented to further study the defects (Fig. 7). Common defects in silica or quartz include oxygen deficiency-related defects and oxygen excess-related defects. Usually, silica with either of these defects have g-values in the range of 1.997 to 2.08^35,36,41^. The difference between the spectrum of the 450 °C sample from the 250 °C or 800 °C sample are the appearance of a peak around 2.0036 g in the 450 °C sample, the greater intensity of the 2.074–2.075 g peak in the 450 °C sample and the appearance of a peak around 2.0036 g in the 450 °C sample. The amber coloration may be attributed to the defects associated with the peak appearing at 2.0036 g. This peak could be related to the aluminum-associated hole centers, as an Al^3+^ ion substitutes a Si^3+^ ion, leaving an unpaired electron at one of the four oxygen atoms next to the Al center^39,43^. This localized spin gives rise to an ESR signal and is responsible for the smoky coloration in irradiated α-quartz. According to the EPR study of silicon dioxide, the g-value around 2.08 could be due to the non-bridging oxygen hole center^35^. In our case, the intensity of this hole center varied with different sintering temperatures, which might be due to the thermodynamic preferences of the coordination among silicon, aluminum, and oxygen.

In this work, we report for the first-time experimental observations on the unusual amber coloration of sol-gel prepared amber glass annealed at different temperatures up to 800 °C. In order to understand this phenomenon, we employed different fundamental hypotheses from chemical and structural related changes to temperature-related defect center generation. The latter is currently considered to be the most plausible cause of such unusual coloring behavior. This new material has the potential for applications such as porous encapsulation of molecules and cells with UV and blue light blocking capability. Furthermore, the new insight in defect chemistry of aluminum doped sol-gel silica can be utilized in optoelectronics and possibly photocatalysis.

## Methods

In our work, the sol-gel approach was applied to fabricate the various-colored glasses. 2.5 molar percentage aluminum nitrate precursor (0.094 grams) was added into a mixture of 2.2 mL tetraethyl orthosilicate (TEOS), 1 mL DI H_2_O, and 2 mL Ethanol. A small amount (around 0.5 mL) of 1 M hydrochloric acid is also added into the mixture. The resulting solution was then stirred at room temperature for 1 hour to form the sol. The sol is then left to sit at room temperature for a few days, and a cap completely covers the opening to prevent any air from getting into the solution. After a few days, pin holes are made in the cap to allow for flow into the gel, and it is left to sit for another one to two weeks. Finally, the gel is heated at a rate of 0.5 °C to 60 °C and then at a rate of 1 °C to the final curing temperature.

The pore structure of the glass was determined using BET (Tristar II 3020, Surface Area Analyzer), Scanning Electron Microscopy (FEI QUANTA 600 F environmental SEM) and Transmission Electron Microscopy (FEI TITAN 80–200 TEM/STEM). The optical property was measured using UV-Vis spectrometer with a 10 mm integrated sphere (JASCO V-670). The elemental composition and chemical bonding were characterized using X-ray Photoelectron Spectroscopy (ThermoScientific ESCALAB 250 XPS). The pair distribution function (PDF) experiment was carried out at the 11 ID-C stations of the Advanced Phonon Source (APS), Argonne National Laboratory (ANL). A focused monochromatic X-ray beam about 5 μm in diameter (FWHM) with a wavelength of 0.4066 Å was used for the diffraction experiments. A MAR345 image plate recorded the diffraction data, and then the two-dimensional (2D) images were integrated to one-dimensional (1D) patterns with the Fit2D program. Si, Al and O K-edge X-ray absorption spectroscopy (XAS) measurements were conducted at the bending magnet beamline 6.3.1 of the Advanced Light Source (ALS), Lawrence Berkeley National Laboratory (LBNL) with an electron energy of 1.9 GeV and a current of 500 mA. Total electron yield mode was used. Electron paramagnetic resonance (EPR) measurements were performed by the Bruker Elexsys E 500 spectrometer with a frequency of 100 kHz. The signals of the defects were observed with a microwave power of 10 mW at 77 K.

## Supplementary information

Supplementary Info

## Data Availability

The datasets generated during and/or analyzed during the current study are available from the corresponding author on reasonable request.
