# Supplementary material for: On the unusual amber coloration of nanoporous sol-gel processed Al-doped silica glass: An experimental study
Source: Sci Rep. 2019 Aug 28;9:12474. doi: 10.1038/s41598-019-48917-4 (PMC6713779; doi:10.1038/s41598-019-48917-4)

*Supplementary Information – Scientific Reports*

**On the unusual amber coloration of nanoporous sol-gel processed Al-doped silica glass: An experimental study**

A. Chang^a^, Y. He^a^, M. A. Torres Arango^b^, M. Wang^a^, Y. Ren^c^, Z. Feng^a^, C.-H. Chang^a^, and K. A. Sierros^b^

^a^ *School of Chemical, Biological and Environmental Engineering, Oregon State University, Corvallis, OR 97331*

*^b^ Mechanical & Aerospace Engineering, West Virginia University, Morgantown, WV 26506 - 6106*

*^c^ Advanced Photon Source, Argonne National Laboratory, 9700 S Cass Avenue Argonne, IL, 60439*

**Table of Contents**

1. **Sol-Gel Kinetics**
2. **N_2_-BET S2**
3. **SEM S3**
4. **TEM S3**
5. **UV-vis Further Explanations S3**
6. **TGA S4**
7. **Photoluminescent spectra S5**

**1) Sol-Gel Kinetics**

The sol-gel process consists of three main steps. The first step is hydrolysis, where a liquid alkoxide precursor (TEOS, or Si(OC_2_H_5_)_~~4~~_, in this case) is hydrolyzed by mixing with water.

Hydrolysis: Si(OC_2_H_5_)_~~4~~_ + 4(H_2_O) 🡪 Si(OH)_4_ + 4(C_2_H_5_OH)

OC_2_H_5_ OH

H_5_C_2_O-Si-OC_2_H_5_ + 4(H_2_O) 🡪 HO-Si-OH + 4(C_2_H_5_OH)

OC_2_H_5_ OH

Next, the hydrated silica tetrahedra will interact with each other in a condensation reaction to form siloxane bonds.

Condensation: Si(OH)_4_ + Si(OH)_4_ 🡪 Si(OH)_3_-O-Si(OH)_3_ + H_2_O

OH OH OH OH

HO-Si-OH + HO-Si-OH 🡪 OH-Si-O-Si-OH + H_2_O

OH OH OH OH

Finally, further linkage of silica tetrahedra will occur in a polycondensation reaction. The water and alcohol formed from the reaction remain the porous gel network.

Polycondensation:


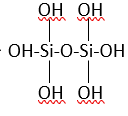


🡪

6Si(OH)_4_

+

HO HO

HO-Si-OH HO-Si-OH

HO O O HO

HO--Si--O—Si----O----Si—O—Si—OH

HO O O HO

HO-Si-OH HO-Si-OH

HO HO

**2) N_2_-BET**

N_2_-BET measurements were taken by breaking off approximately 0.245 grams of the sample. The sample was not crushed and was degassed and tested. Microporosity analysis (by monitoring nitrogen gas absorption) was performed with an ASAP2020 accelerated surface area and porosity system Micromeritics®. Degassing of samples for microporosity measurements was performed using a Micromeritics vac-prep system for 4.5 days at 105°C, and 1 h at 210°C, followed by cooling to room temperature in 30 min.

**3) SEM**

The samples for the SEM images in Figure 3 were first etched by 0.01 M sodium hydroxide for 3 minutes and then set on carbon tapes. A thin film of gold-platinum was coated on the samples by the Cressington Au-Pd coater.

**4) TEM**

TEM samples were prepared by grinding the glass in an agate mortar while using ethanol. The pulverized sample was transferred to a clean glass vial, and more ethanol was added, forming a turbid amber and white solution. Such solution was sonicated for ~ 5 min. A droplet of the solution was placed on an ultrathin carbon TEM grid (TED Pella) and allowed to dry. TEM imaging was performed with a JEOL JEM-2100 TEM at 200kV acceleration voltage equipped with a Gatan Erlangshen ES500W digital camera and a Gatan Orius SC600 high-resolution digital.

1. **UV-Vis Further Explanations**



The transmittance higher than 100 % in the UV region can be ascribed to the photoluminescent phenomenon of the 800 °C sample. The photomultiplier is the photon detector in the JASCO UV-Vis 670, which was applied to obtain the transmittance and absorption coefficient in this manuscript. This kind of light detector usually counts the number of photons but cannot recognize the energy of each photon. In this case, if the sample is photoluminescent, both its transmitted and emitted photons are detected by the detector simultaneously in the excitation region.

Additionally, the detector has different sensitivities of the UV photons and visible photons (emitted photons). Usually, this difference can be leveled by using the background spectrum. However, since both UV and visible photons were detected at the same time, the higher sensitivity of the visible photons resulted in a transmission higher than 100 % and an absorption coefficient lower than 0.

**6) TGA**


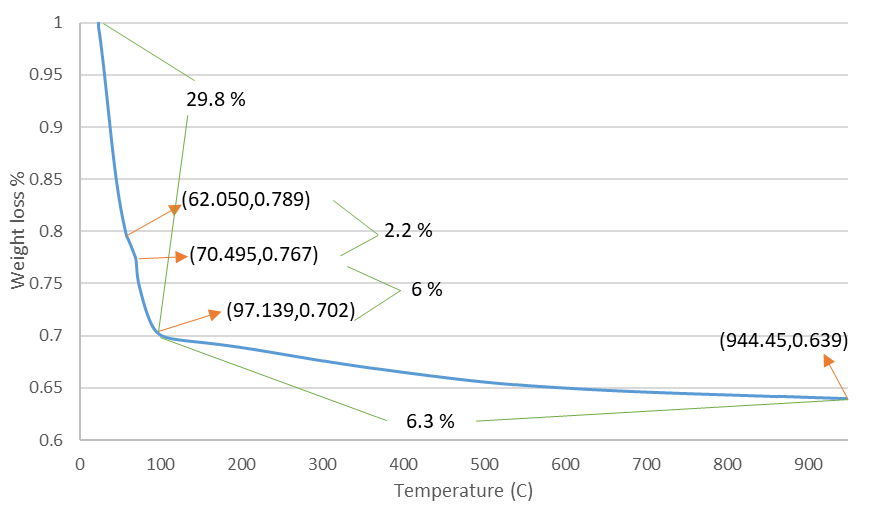


TGA Q600 data measurements were taken by breaking off approximately 0.15 grams of the sample. The samples were then heated at a ramp from room temperature to 60 ^o^C at a rate of 0.5 ^o^C/min, and then from 60 ^o^C to 650-850 ^o^C at a rate of 1 ^o^C/min.

TGA weight loss measurements indicate that 29.8% of the total solution weight is lost by 100**°** C. We hypothesize that this initial large weight loss is due to the vaporization of the excess ethanol and water in the solution. There is also continued weight loss after 100**°** C until the final annealing temperature, which results in an additional 6.3% of the solution weight being lost. We attribute this additional weight loss to the decomposition or combustion of any ethyl groups left in the silica gel network as not all of the ethyl groups will participate in the hydrolysis reaction.

**7) Photoluminescent Spectra**

(a) Photoluminescent emission spectra under 250nm light. (b) Photoluminescent excitation spectra with an emission wavelength at 525 nm.

The spectra show a peak at 525 nm which can be seen from the 450°C and 800°C samples. Photoluminescent excitation spectra with emission at 525nm are also shown on the right (b). In these spectra, the 800°C sample shows a higher excitation from 200nm to 250nm which is consistent with the transmittance spectrum (Figure 2 in the 1^st^ version of the manuscript) in which 800°C has the transmittance higher than 100% resulting from the PL. The peaks at 500 nm (Figure a) and 262.5 nm (Figure b) are the background luminescence due to the excitation light and emission detector which has a wavelength of 250 nm and 525 nm, respectively.


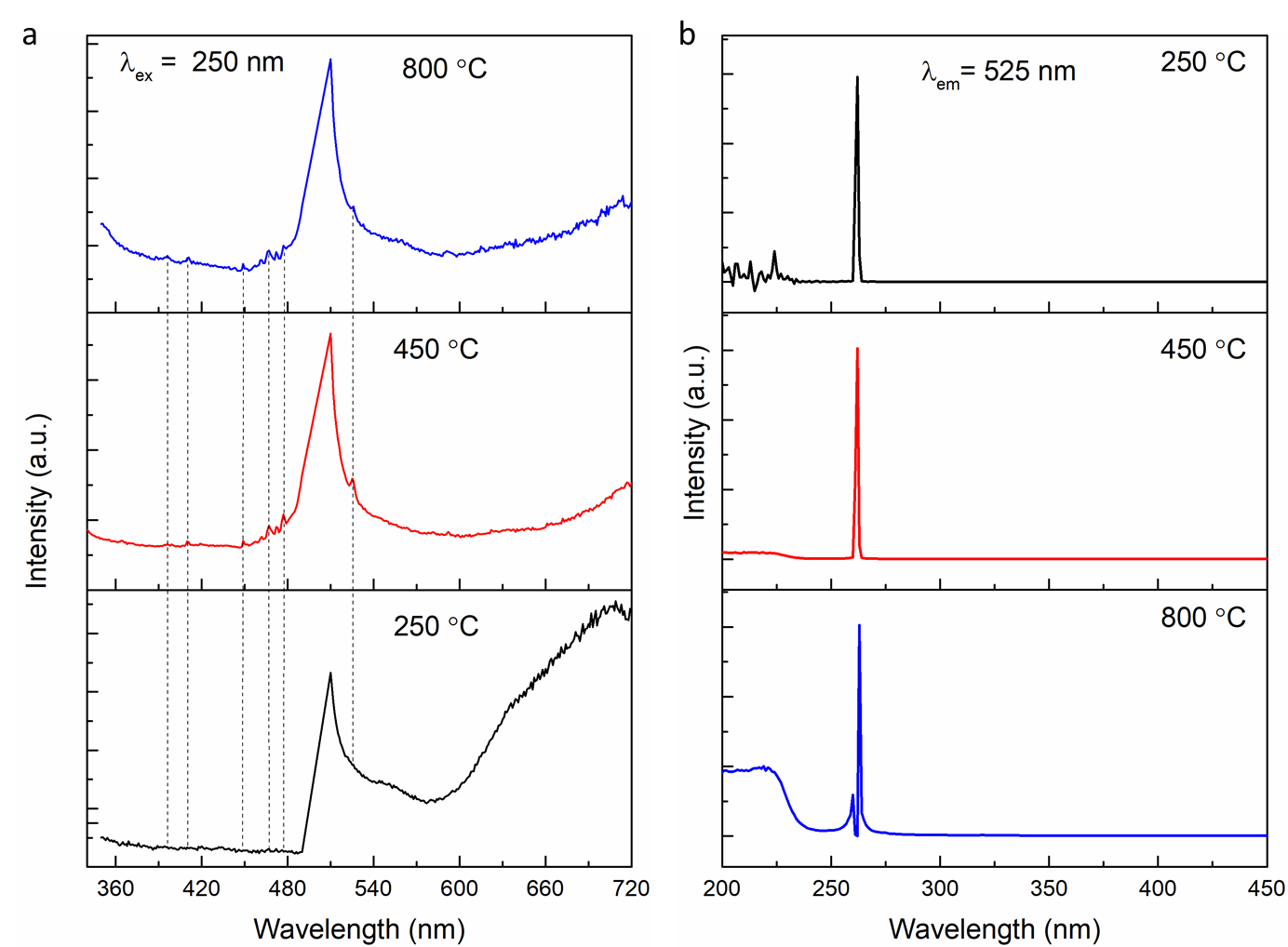

Supplement: Supplementary file 1 — Supplementary Info [file 41598_2019_48917_MOESM1_ESM.docx]
